# Paneth-cell-disruption-induced necrotizing enterocolitis in mice requires live bacteria and occurs independently of TLR4 signaling

**DOI:** 10.1242/dmm.028589

**Published:** 2017-06-01

**Authors:** Jessica R. White, Huiyu Gong, Brock Pope, Patrick Schlievert, Steven J. McElroy

**Affiliations:** 1 Pediatrics, University of Iowa, Iowa City, IA 54424, USA; 2 Microbiology, University of Iowa, Iowa City, IA 54424, USA

**Keywords:** Necrotizing enterocolitis, Paneth cells, Epithelial barrier, Enterocyte biology, Mucosal defense, Mice

## Abstract

Necrotizing enterocolitis (NEC) remains a leading cause of morbidity and mortality in premature infants. Both human surgical specimens and animal models suggest a potential involvement of Paneth cells in NEC pathogenesis. Paneth cells play critical roles in epithelial homeostasis, innate immunity and host-microbial interactions. Yet, the complex interplay between Paneth cell disruption, epithelial barrier dysfunction and microbial-driven inflammation remains unclear in the immature intestine. In this study, mucosal intestinal injury consistent with human NEC was induced in postnatal day 14-16 (P14-P16) mice by disrupting Paneth cells, followed by gavage with *Klebsiella pneumonia.* Mucosal injury was determined by histology, serum cytokine levels and epithelial barrier dysfunction. Toll-like receptor 4 (TLR4) activation was examined using protein expression, gene expression, and *TLR4*^−/−^ mice. Finally, the role of bacteria was evaluated using heat-killed bacteria, conditioned media, *Bacillus cereus* and cecal slurries. We found that live bacteria were required to induce injury; however, TLR4 activation was not required. NEC induced by Paneth cell disruption results in altered localization of tight junction proteins and subsequent loss of barrier function. Prior research has shown a requirement for TLR4 activation to induce NEC-like damage. However, many infants develop NEC in the absence of Gram-negative rod bacteremia, raising the possibility that alternative pathways to intestinal injury exist. In this study, we show a previously unknown mechanism for the development of intestinal injury equivalent to that seen in human NEC and that is not dependent on TLR4 pathways. These data are congruent with the new hypothesis that NEC may be the consequence of several disease processes ending in a final common inflammatory pathway.

## Abstract (precis)

**Summary:** Paneth cell disruption and bacterial exposure in immature murine intestine induces NEC-like pathology. Importantly, this occurs independently of TLR4 activation, presenting an alternative mechanism for disease development.

## INTRODUCTION

Necrotizing enterocolitis (NEC) is a devastating gastrointestinal disease found almost exclusively in premature infants (Minino et al., 2002). The pathogenesis of NEC is presumed to involve translocation of bacteria across immature defense barriers of the intestinal epithelium, leading to tissue invasion and damage. This mechanism is supported by clinical signs of NEC. These include pneumatosis intestinalis [gas pockets within the bowel wall caused by bacterial fermentation (Hill et al., 1974)], epidemic episodes of NEC, and infant bacteremia or endotoxemia (Bizzarro et al., 2014). In addition, several studies have reported an increase in activation of Toll-like receptor 4 (TLR4) in infants who have developed NEC (Leaphart et al., 2007; Neal et al., 2013), and disruption of TLR4 genes have been associated with NEC development (Sampath et al., 2011, 2015). TLR4 activation has also been found to be necessary for the induction of NEC by formula feeding and systemic hypoxia (hypoxia/formula) in rodents (Jilling et al., 2006; Leaphart et al., 2007; Chan et al., 2009). TLR4 activation is a key mechanism by which the host can detect and respond to the lipopolysaccharide contained in the cell wall of Gram-negative bacteria. However, many infants develop disease without signs of Gram-negative invasion, raising the possibility of additional mechanisms to induce NEC.

Another important mechanism by which humans regulate their intestinal bacterial composition is through the activity of Paneth cells (Salzman et al., 2010; Bevins and Salzman, 2011). Paneth cells, located in the intestinal crypts of the ileum, secrete a host of cytokines and antimicrobial peptides to directly impact the homeostasis of the stem cell niche and the host-microbial axis (Bevins and Salzman, 2011; Clevers and Bevins, 2013). Our lab and others have previously shown that infants who developed NEC had significantly fewer Paneth cells than controls (Coutinho et al., 1998; McElroy et al., 2011). Since Paneth cells directly affect the composition of intestinal bacteria, it is reasonable to hypothesize that TLR4 is involved in Paneth-cell-disruption-induced injury in the immature intestinal tract. However, studies that mechanistically link Paneth cells and TLR4 have not been previously performed in regard to NEC because the majority of animal models of NEC either do not contain Paneth cell lineages (piglet) or are performed in animals prior to normal ontological development of Paneth cells (hypoxia/formula rodent models) (Bry et al., 1994). To address this gap in knowledge, we utilized our Paneth-cell-disruption model of NEC (Zhang et al., 2012; McElroy et al., 2014) to investigate the role of TLR4 activity in the development of intestinal injury.

Our hypothesis for this study was that TLR4 signaling would be required to induce injury in our Paneth-cell-disruption model. Our results revealed an injury pattern consistent with clinical diagnosis of human NEC; however, this injury occurred in the absence of TLR4 signaling. These data are noteworthy as NEC often occurs in premature infants without the presence of a Gram-negative rod in the infant's blood, peritoneal or tissue cultures. Furthermore, NEC is now hypothesized to be a common phenotypic endpoint to several mechanistic processes (Gordon et al., 2012). Thus, our data support the presence of a previously unidentified mechanism to develop NEC-like pathology in the absence of TLR4.

## RESULTS

### Paneth-cell-disruption-induced injury is similar to the pathophysiology seen in human NEC

**Fig. 1.**
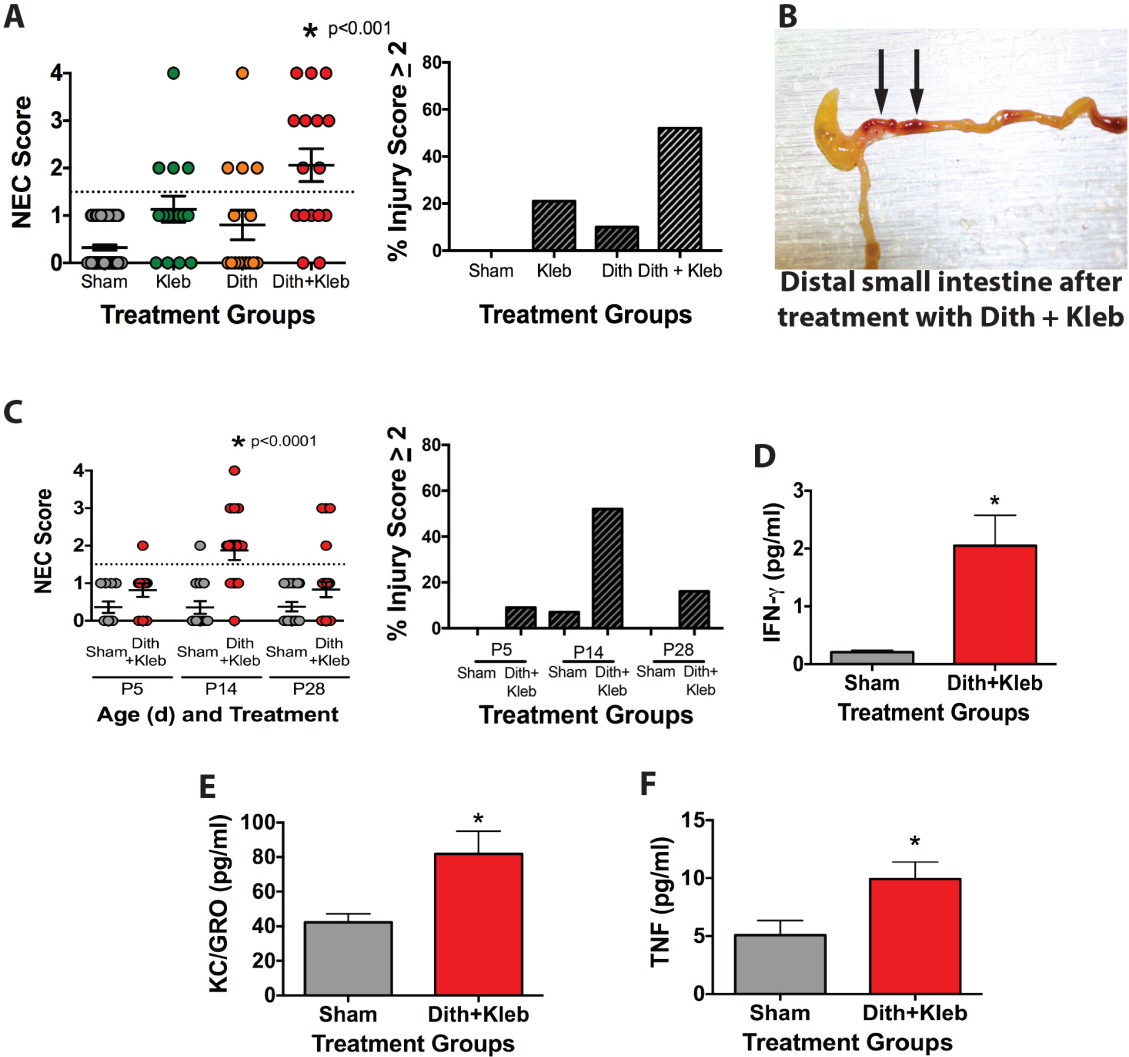
**Paneth-cell-disruption-induced NEC shows characteristics consistent with development of human NEC.** (A) Mice receiving an intraperitoneal injection of dithizone followed by gavage of *Klebsiella* consistently develop injury (score of 2 or greater, represented by those lying above the dotted line), compared to sham mice, mice receiving *Klebsiella* alone, or mice receiving dithizone alone (*P*<0.001, *n*=16 for treatment groups, 71 for sham). (B) Patchy necrotic areas (arrows) are visualized on the ileum of mice receiving dithizone followed by *Klebsiella*. (C) P5 mice, prior to the development of Paneth cells, do not exhibit intestinal injury (*P*=0.40, *n*=11 for each group). P28 mice also do not develop significant intestinal injury (*P*=0.52, *n*=17 sham and 25 treatment). Only P14-P16 mice develop significant intestinal injury (*P*<0.001, *n*=14 sham and 16 treatment). (D) Mice treated with dithizone followed by *Klebsiella* exhibited significant increases in serum IFNγ, (E) KC/GRO (the murine homolog of IL-8; *P*=0.0091, *n*=21 sham, 29 treatment), and (F) TNF compared to sham controls (*P*=0.0021, *n*=21 sham, 29 treatment).

**Fig. 2.**
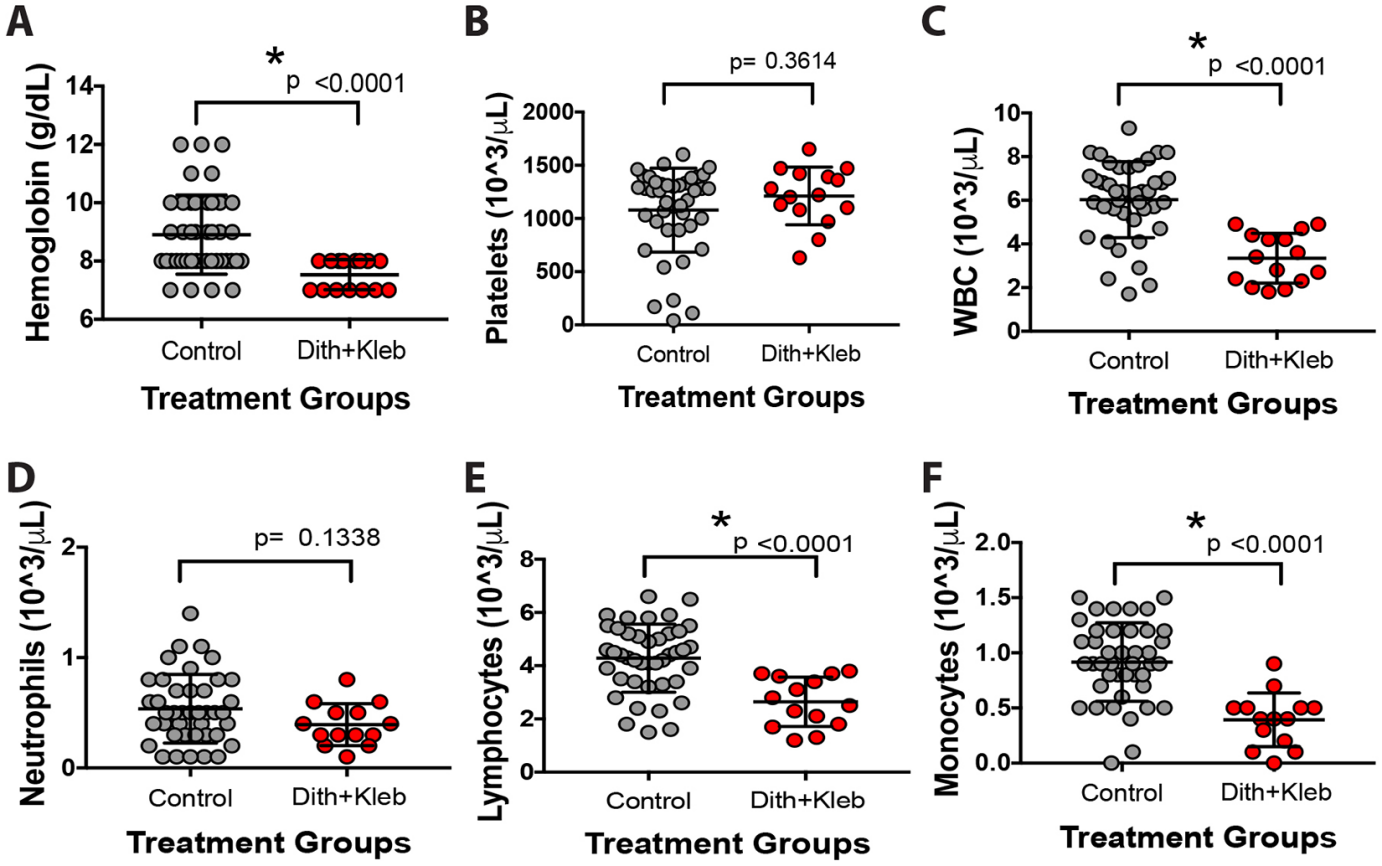
**Paneth cell disruption induces anemia and leukopenia.** Serum samples were collected, diluted 10-fold and placed on ice until analysis (<30 min from sampling). Analysis was performed using a Sysmex XT-200iV analyzer in manual capillary mode. P14-P16 mice exposed to dithizone followed by *Klebsiella* (*n*=42) were compared to control animals (*n*=15) and were found to have significantly lower hemoglobins (A; 7.5 vs 8.9 g/dl, *P*<0.0001), white blood cell (WBC) counts (C; 3.3 vs 6.0×10^3^/µl, *P*<0.0001), absolute lymphocyte counts (E; 2.6 vs 4.3×10^3^/µl, *P*<0.0001) and absolute monocyte counts (F; 0.4 vs 0.9×10^3^/µl, *P*<0.0001). Platelet counts (B) and absolute neutrophil counts (D) were statistically similar to controls.

The Paneth-cell-disruption model of NEC was originally described in CD-1 mice (Zhang et al., 2012). However, the C57BL/6 strain is more commonly utilized due to its frequent use as the background for genetically modified mice. Because of this, we desired to determine whether the injury pattern in C57BL/6J mice was similar to that of CD-1 mice and human disease. C57BL/6J mice exposed to dithizone and *Klebsiella pneumoniae* as previously described developed intestinal injury consistent with human NEC and equivalent to our prior data using the Paneth-cell-disruption model in CD-1 mice (Fig. 1). In addition to developing NEC-like injury that is specific to the distal small intestine (Fig. 1B), injury induced by Paneth cell disruption also exhibits developmental dependence. When exposed to dithizone and *K.* *pneumoniae*, neither postnatal day 5 (P5) mice (prior to normal ontogenic development of Paneth cells) nor P28 mice (mature intestinal tract) developed significant intestinal injury compared to controls. In contrast, P14-P16 mice (immature intestine but containing Paneth cells) exhibited significant intestinal injury consistent with NEC (*P*<0.0001; Fig. 1C). To further determine systemic effects, we compared serum samples from mice exposed to both dithizone and *K.* *pneumoniae* to control animals for quantification and evaluation of hematologic variables (Fig. 2). Mice exposed to Paneth-cell-disruption-induced NEC had significantly lower hemoglobin (7.5 vs 8.9 g/dl, *P*<0.0001), a significantly lower white blood cell count (3.3 vs 6.0×10^3^/µ, *P*<0.0001), and significantly lower absolute lymphocyte and monocyte counts (2.6 vs 4.3×10^3^/µl, *P*<0.0001 and 0.4 vs 0.9×10^3^/µl, *P*<0.0001) compared to controls.

### Paneth-cell-disruption-induced NEC occurs independently of TLR4 activation or upregulation

**Fig. 3.**
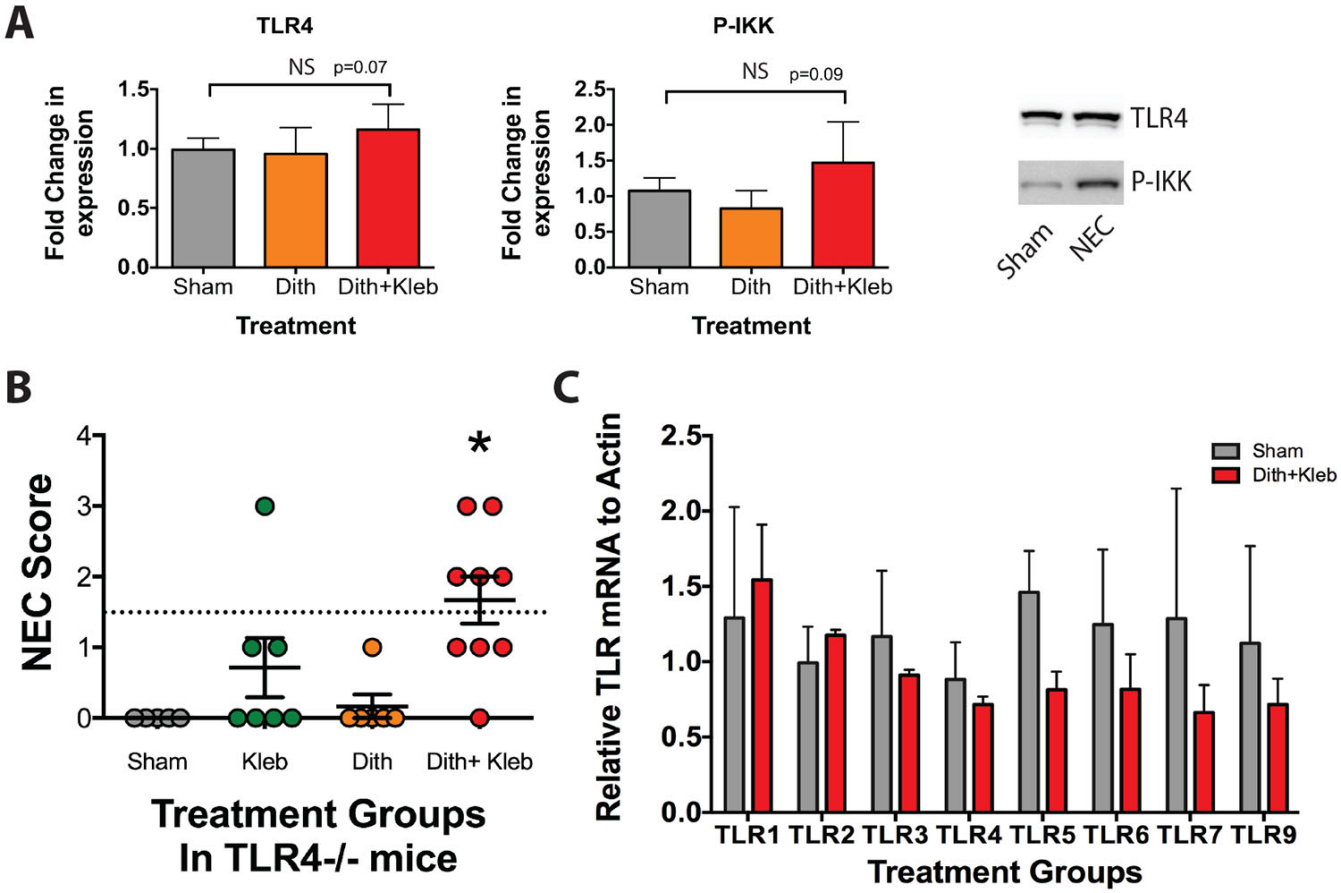
**Development of intestinal injury is not dependent on TLR4.** (A) Paneth-cell-disruption-induced NEC has no significant effect on protein expression of TLR4 or its downstream target pIKK (*P*=0.07 and 0.09 respectively, *n*=10 sham, 11 dithizone and 5 dithizone+*Klebsiella*). A representative western blot is shown to the right. (B) *TLR4*^−/−^ mice develop significant intestinal injury compared to sham when exposed to Paneth-cell-disruption-induced NEC (*P=0.005, *n*=9). (C) Paneth-cell-disruption-induced NEC had no significant effect on gene expression of TLR1-TLR7 or TLR9 (*n*=3).

Previous studies using the hypoxia/hypothermia/formula induction model of NEC have shown a requirement for TLR4 and its downstream signaling pathways to develop injury. To determine whether TLR4 was required in our Paneth-cell-disruption-induced injury model, we began by quantifying the protein levels of TLR4 and its downstream target pIKK in homogenized ileal tissues. Animals exposed to dithizone and *K. pneumoniae* exhibited no significant increase in protein expression of TLR4 or pIKK compared to controls (Fig. 3A).

We next utilized a constitutive *TLR4*^−/−^ mouse in place of the wild type, C57BL/6J. Despite a lack of TLR4, these mice developed significant injury when exposed to dithizone and *K. pneumoniae* (Fig. 3B). Having found that TLR4 signaling was not upregulated or required in the Paneth-cell-disruption model of NEC, we next examined whether other Toll-like receptors (TLRs) were upregulated. Ileal mRNA was quantified for TLR1, 2, 3, 4, 5, 6, 7 and 9 using quantitative real-time reverse transcription-polymerase chain reaction (qRT-PCR) techniques. Induction of Paneth-cell-disruption-induced NEC had no significant effect on gene expression of any TLR that was measured (Fig. 3C).

### Induction of NEC-like injury requires Paneth cell disruption, but does not require dithizone

**Fig. 4.**
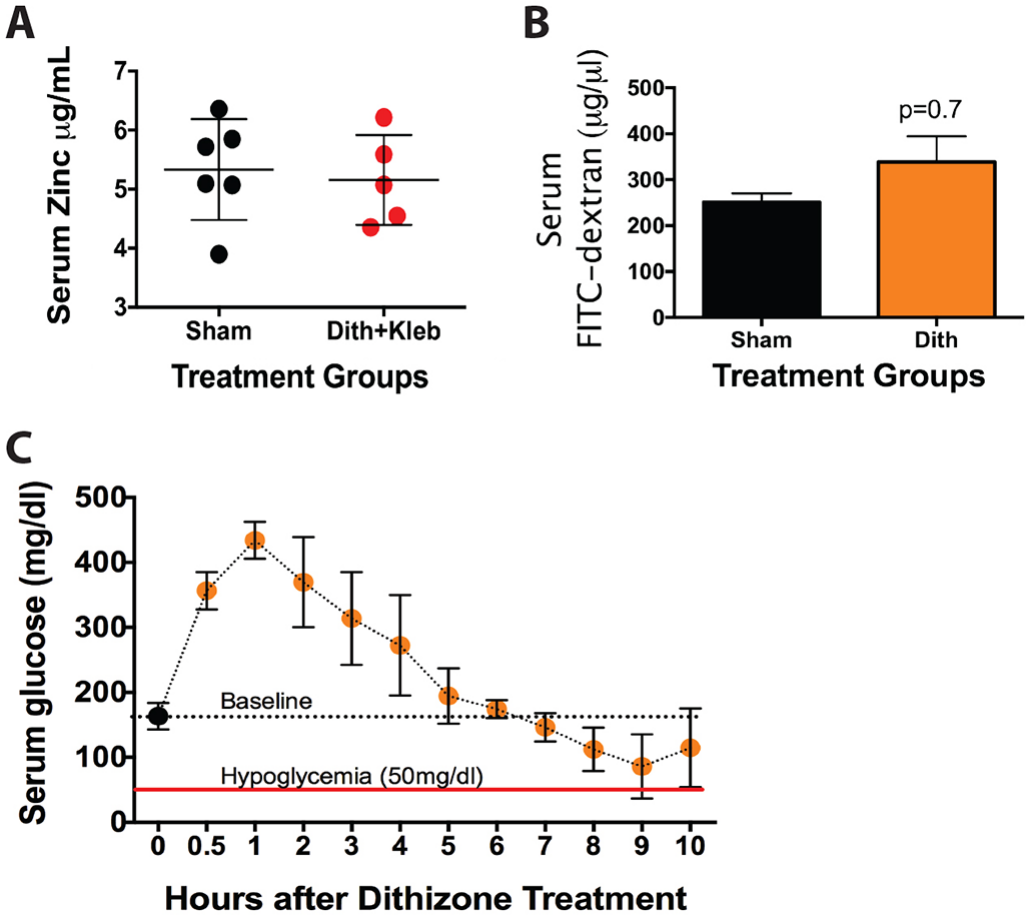
**Intraperitoneal dithizone treatment induces minimal systemic side effects.** P14-P16 mice treated with dithizone have similar (A) serum zinc levels (*n*=5, *P*=0.57) and (B) intestinal epithelia barrier function as determined by FITC-dextran passage (*n*=8, *P*=0.7) compared to sham controls. In addition, dithizone treatment does not induce significant hypoglycemia [*n*=5 per time point, hypoglycemia was defined as 50 mg/dl (Danneman et al., 2013)] and is denoted by the red line.

Dithizone is a heavy-metal chelator that reacts with the zinc contained in Paneth cells to produce zinc-dithizonate complexes and subsequent Paneth cell disruption (Sawada et al., 1991, 1993). While zinc is abundant in Paneth cells it is also a key heavy metal in many biological processes and plays a key role in both glucose homeostasis and tight junction regulation. To confirm that our dose of dithizone was not inducing significant side effects, we tested the effect of dithizone exposure on serum zinc levels (Fig. 4A), intestinal barrier function (Fig. 4B) and serum glucose levels (Fig. 4C). Despite inducing significant loss of Paneth cells, dithizone exposure induced no significant systemic changes from baseline.

**Fig. 5.**
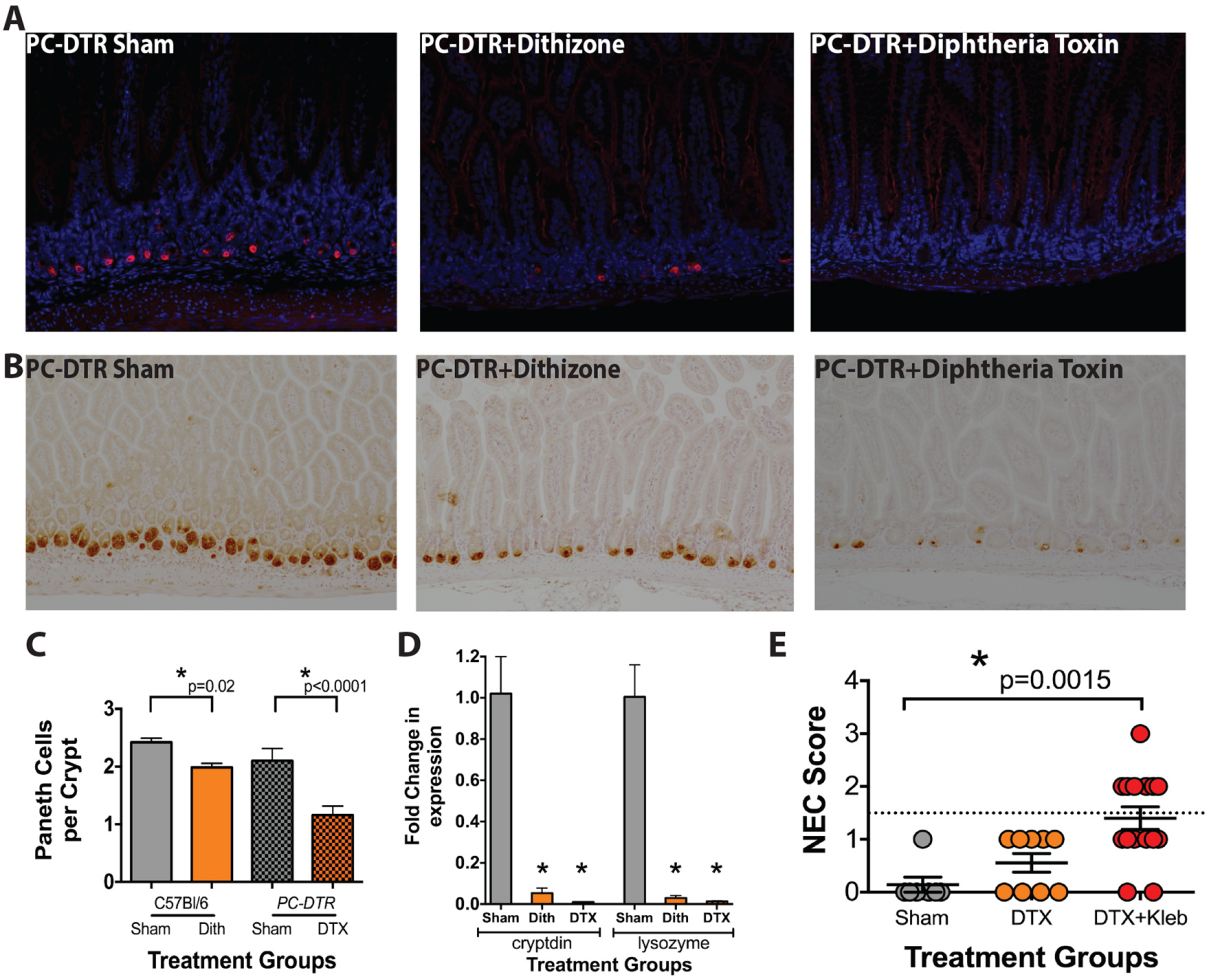
**Paneth-cell-disruption-induced NEC is not dithizone dependent.** *PC-DTR* mice treated with either dithizone or diphtheria toxin (without addition of bacteria) have decreased staining of (A) HA and (B) lysozyme. (A) Red is anti-HA, blue is anti-DAPI. (C) *PC-DTR* mice have decreased Paneth cells per crypt after dithizone or diphtheria toxin (DTX) treatments (*P*=0.02 and <0.001 respectively, *n*≥10). (D) *PC-DTR* mice also have significantly decreased gene expression of cryptdin and lysozyme after dithizone or DTX treatment (*P<0.001, *n*=3). (E) *PC-DTR* mice treated with intraperitoneal injection of dithizone followed by gavage of *Klebsiella* consistently develop injury (score of 2 or greater; represented by those lying above the dotted line), as compared to sham mice (*P*=0.0015, *n*=7 sham, 9 dithizone, 15 dithizone+*Klebsiella*).

To assess whether dithizone itself rather than Paneth cell disruption contributes to injury development, we generated a mouse (*PC-DTR*) with a hemagglutinin (HA)-tagged human diphtheria toxin receptor expressed in Paneth cells via the Paneth-cell-specific-cryptdin-2 (*Defa6*) promoter (Garabedian et al., 1997). Staining for the HA tag (Fig. 5A) and lysozyme (Fig. 5B) demonstrated that Paneth cells were present in *PC-DTR* control mice, and that our construct was Paneth-cell-specific. Treatment with diphtheria toxin induced significant decreases in the quantity of granule-positive Paneth cells per crypt and in the expression of both of the Paneth cell markers, cryptdin and lysozyme, compared to controls (Fig. 5C,D). Lastly, similar to our findings using dithizone, *PC-DTR* mice given diphtheria toxin followed by a gavage of *K.* *pneumoniae* produced significant ileal injury that was histopathologically similar to that seen in human NEC (*P*=0.026; Fig. 5E).

### Development of NEC-like injury requires live bacteria, but does not require Gram-negative rods

**Fig. 6.**
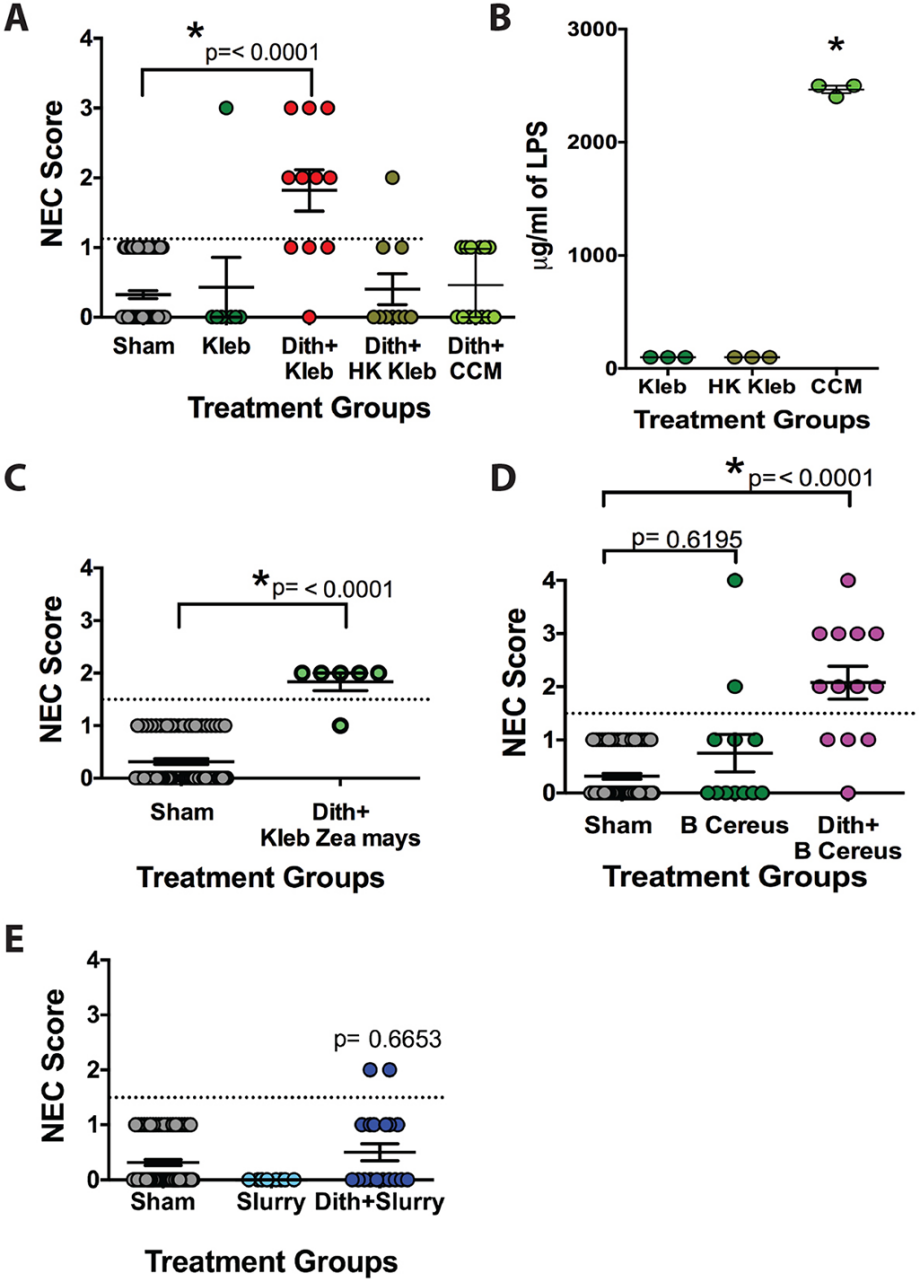
**Paneth-cell-disruption-induced NEC requires live bacteria but is not dependent on Gram-negative strains.** (A) Mice receiving heat-killed (HK) *Klebsiella* (*n*=10) or condensed, conditioned media (CCM; *n*=13) substituted for live *Klebsiella* showed similar injury scores compared to sham controls. (B) LPS levels of heat-killed *Klebsiella* were equivalent to live bacteria (*n*=3) and LPS levels of condensed, conditioned media were 24 times greater than live *Klebsiella* (*n*=3, mean of 2467 pg/ml compared to 100 pg/ml, *P=<0.0001). (C) Mice receiving *Klebsiella* *Zea mays* substituted for *K.* *pneumoniae* ATCC10031 following dithizone treatment had significant intestinal injury compared to shams and similar to that following dithizone injection and gavage of *K.* *pneumoniae* ATCC10031 (*n*=6, *P*<0.0001). (D) Mice receiving *B.* *cereus* substituted for *K.* *pneumoniae* ATCC10031 following dithizone treatment had significant intestinal injury compared to sham controls and similar to that following dithizone injection and gavage of  *K.* *pneumoniae* ATCC10031 (*n*=13, *P*<0.0001). (E) Mice receiving a cecal slurry gavage substituted for *K.* *pneumoniae* ATCC10031 following dithizone treatment had a lack of intestinal injury similar to sham (*n*=20, *P*=0.6653).

Our model of NEC requires both Paneth cell disruption and exposure to bacteria in order to induce injury. Since our data show that TLR signaling is not critical to development of intestinal injury, we next aimed to determine whether live bacteria were required, or if bacterial components suffice. We used either heat-killed *K. pneumoniae*, or condensed, conditioned media in place of live *K. pneumoniae.* Neither bacterial substitute was able to produce significant intestinal injury within our model compared to controls (Fig. 6A). This absence of injury was not due to lipopolysaccharide (LPS) concentrations: LPS concentrations in heat-killed and live bacterial samples were similar, and LPS concentrations of condensed, conditioned media were over 20 times that of live bacteria (Fig. 6B).

To determine whether intestinal injury in the Paneth-cell-disruption model required a specific live strain of *Klebsiella*, we substituted *Klebsiella Zea mays*, a green fluorescent protein (GFP)-tagged wild-type strain of *K. pneumoniae* that is found associated with the stem tissue of *Zea mays* (Chelius and Triplett, 2000), for *K.* *pneumoniae* ATCC10031. Mice receiving *Klebsiella Zea mays* also developed significant NEC compared to controls (Fig. 6C) and had an injury profile similar to mice receiving *Klebsiella* ATCC10031. To determine whether members of the *Klebsiella* genus were required for development of injury, we next substituted *K.* *pneumoniae* with the Gram-positive bacteria *Bacillus cereus*. Mice receiving *B.* *cereus* gavage in place of *K. pneumoniae* also developed significant injury compared to controls, and similar injury compared to mice receiving *Klebsiella* ATCC10031 (Fig. 6D). Lastly, to determine whether non-native bacteria were required for injury development, we used cecal slurries in place of *K.* *pneumoniae*. Neither mice receiving gavage of cecal slurries alone nor mice receiving gavage of cecal slurry following Paneth cell disruption with dithizone developed significant injury compared to controls (Fig. 6E).

### Paneth-cell-disruption-induced NEC causes significant epithelial barrier dysfunction

**Fig. 7.**
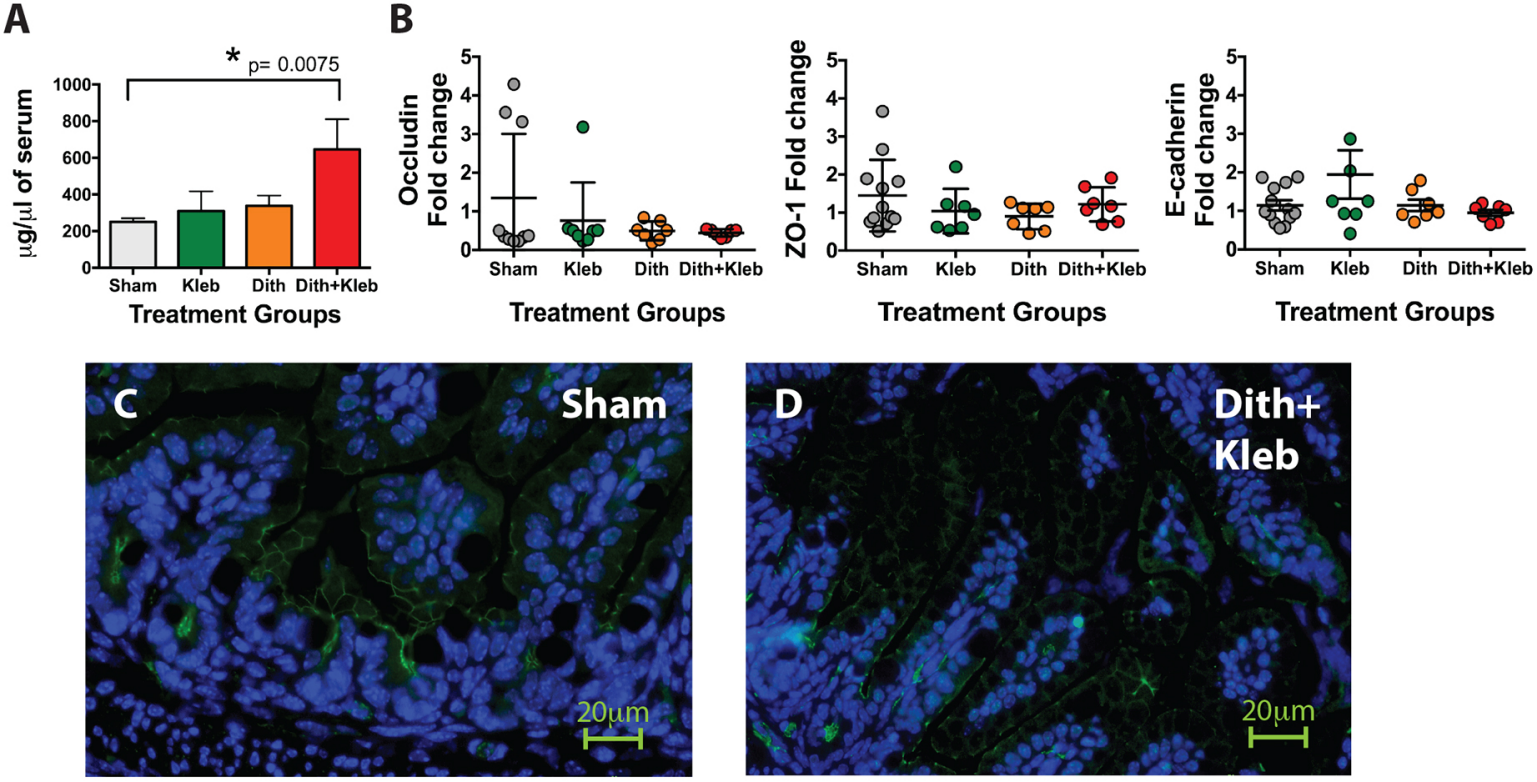
**Paneth-cell-disruption-induced NEC induces epithelial barrier dysfunction in addition to tissue injury.** (A) Paneth-cell-disruption-induced NEC significantly increases epithelial barrier dysfunction as measured by translocation of fluorescein isothiocyanate (FITC)-dextran (measured in μg) into the serum (*n*=7, *P*=0.0075). (B) Paneth-cell-disruption-induced NEC has no effect on zona occludens 1 (*ZO-1*), occludin or E-cadherin gene expression (*n*≥7 for each experimental group). (C,D) Normal ZO-1 localization at the villus tips (C) is disrupted by Paneth-cell-disruption-induced NEC (D). ZO-1 staining is shown in green with nuclear staining (DAPI) in blue. *n*=5 animals per condition with 3-4 areas examined per animal; representative histology shown.

A key hallmark of NEC is disruption of the intestinal epithelial barrier. To determine whether Paneth-cell-disruption-induced NEC similarly altered intestinal barrier function, we used fluorescein isothiocyanate (FITC)-dextran as a marker of paracellular transport and mucosal barrier dysfunction. After exposure to dithizone and *Klebsiella*, C57BL/6J mice were gavaged with FITC-dextran. At 4 h after gavage, serum samples were obtained and FITC-dextran levels were compared to controls. Only animals exposed to both Paneth cell disruption and *Klebsiella* showed significant barrier dysfunction (Fig. 7A). Neither zona occludens 1 (*ZO-1*) nor E-cadherin mRNA expression was altered by Paneth-cell-disruption-induced NEC, compared to controls (Fig. 7B). However, mice with NEC-like injury (Fig. 7D) have a decreased expression of ZO-1 at the villus tips compared to controls (Fig. 7C), suggesting an alteration in the localization of tight junction proteins, which concurs with previous investigations (Clark et al., 2006).

## DISCUSSION

NEC remains the major cause of gastrointestinal morbidity and mortality in preterm infants. Although the pathophysiology of NEC remains incompletely understood, a widely held hypothesis involves activation of TLR4 signaling by the LPS contained in the cell wall of Gram-negative bacteria (Hackam et al., 2013; Sodhi et al., 2015). Disruption of Paneth cells has also been implicated as a possible trigger for development of NEC (McElroy et al., 2011, 2013; Zhang et al., 2012) and, since Paneth cells play a role in regulating the composition of the intestinal flora, it is reasonable that these two pathways are complementary. Although we are beginning to understand that Paneth cells are critical to the health and homeostasis of the adult small intestine (Clevers and Bevins, 2013), their role in the immature intestine remains less clear. Here, we show that Paneth cell disruption followed by enteral bacterial exposure can induce small-intestinal injury in C57BL/6J mice that is similar to the intestinal injury seen in infants with NEC. While live bacteria are necessary, they do not have to be Gram-negative rods and TLR4 signaling is not required for development of intestinal injury.

Our model is a two-hit model requiring both Paneth cell disruption and exposure to enteral bacteria. Paneth cells are complex columnar intestinal epithelial cells that are located in the intestinal crypts of Lieberkühn and reside between intestinal epithelial stem cells. These cells are unique among the intestinal epithelial cells due to their cytoplasmic granules, which contain cytokines and anti-microbial peptides that are secreted constitutively and in response to bacterial antigens. Paneth cell secretions help to maintain a semi-sterile intestinal crypt niche and regulate the intestinal microbiome (Clevers and Bevins, 2013). Paneth cells are also central to regulating mucosal development, providing key cellular factors critical to maintenance of the intestinal stem cell niche, and providing host defense (Bevins and Salzman, 2011; Clevers, 2013; Sailaja et al., 2016). However, the exact role of Paneth cells remains unclear. While intestinal stem cells grow better in the presence of Paneth cells (Sato et al., 2009, 2011), ablation of Paneth cells in intestinal organoids has been shown to have no effect on the number or functional of stem cells (Durand et al., 2012; Kim et al., 2012). However, understanding the role of Paneth cells in the immature intestine is much more complex than in adult tissue as the developing intestine contains fewer Paneth cells and those that are present may not function as well as those contained in mature intestine (Heida et al., 2016). Thus, understanding the effects of Paneth cell disruption in the immature intestine may be of critical importance to understanding injury and repair mechanisms. In our model, Paneth cell disruption is induced through treatment with dithizone, a heavy-metal chelator. However, our findings are not dependent on dithizone but rather on the disruption of Paneth cells. Mice exposed to dithizone and *Klebsiella* prior to development of Paneth cells do not develop injury, and Paneth cell disruption utilizing diphtheria toxin with our *PC-DTR* transgenic mice induces injury equivalent to that induced by dithizone treatment.

The second hit in our model is enteral exposure to bacteria. Since the very first descriptions of NEC, clinicians have felt that bacteria were a key determinant in pathogenesis (Mizrahi et al., 1965; Santulli et al., 1975). Recent work has demonstrated a key role for TLR4 in NEC pathogenesis (Jilling et al., 2006; Leaphart et al., 2007; Sodhi et al., 2015; Nino et al., 2016). However, these studies have primarily been performed in animal models prior to normal ontological development of Paneth cells (Bry et al., 1994; Garabedian et al., 1997). Since our model of Paneth-cell-disruption-induced NEC creates a pathology that is similar to historic models of NEC (Zhang et al., 2012) (Fig. 1), we wanted to examine the role that TLR4 played in the development of injury in our model. Counter to our expectations, we found no significant upregulation in either TLR4 or pIKK protein expression following the development of injury. We also found no upregulation in gene expression for any of the TLRs. Since our samples are homogenized tissue, it is possible that significant results may be diluted out by non-epithelial cells. Because of this, we next used *TLR4*^−/−^ mice in our Paneth-cell-disruption model of NEC. Contrary to other models (Jilling et al., 2006; Leaphart et al., 2007; Sodhi et al., 2015), we were able to induce equivalent NEC-like injury even in the absence of TLR4.

TLR4 is a pattern recognition receptor that localizes at the cell surface, and classically ligates with LPS to incite an inflammatory response in the host organism. TLR4 is widely distributed across different species and different cell types, and, importantly in humans and mice, intestinal TLR4 expression is higher in immature tissues than in more mature samples (Gribar et al., 2009; Sodhi et al., 2010; Nino et al., 2016). More than 30 types of polysaccharides have been found to interact either directly or indirectly with TLR4 (Zhang et al., 2016), but the most well known of these is the Gram-negative bacterial-cell-wall glycolipid LPS. The role of TLR4 in NEC is believed to occur when Gram-negative bacteria translocate across the epithelium to interact with TLR4 and induce further inflammation (Lu et al., 2014). While TLR4 clearly plays a role in development of NEC and represents an attractive mechanism, it is likely not the only answer. Most experts now believe that NEC represents a common final pathway originating through several potential mechanisms (Gordon et al., 2012). This helps explain why no single bacteria has been determined to be causative of NEC; in fact, many infants develop NEC without the clinical appearance of Gram-negative sepsis (Clark et al., 2012; Bizzarro et al., 2014; Coggins et al., 2015).

In summary, the Paneth-cell-disruption model of NEC requires Paneth cell disruption and not specifically dithizone exposure or lack of Paneth cells to induce injury. Additionally, we have shown that the model requires live, non-native bacteria to induce injury, but the bacteria do not have to be Gram-negative rods. Furthermore, we show that TLR4 activation is not required to induce injury. Our model significantly differs mechanistically from other models of NEC and yet develops a pathophysiology that is similar both to animal models and human NEC, supporting the hypothesis that clinical NEC is a final common pathway originating from several causes (Gordon et al., 2012). We note that NEC in humans is multifactorial and that, like all rodent models, ours does not necessarily recapitulate all facets of the disease in all patients. However, based on our results, we propose that Paneth cell disruption followed by enteral exposure to dysbiosis can be one pathway to developing intestinal injury in the immature gut. This is extremely clinically relevant. Infants most often develop NEC between 27 and 34 weeks corrected gestation (Yee et al., 2012; Stoll et al., 2015), an age when the infant's intestinal tract has a relatively hyper-reactive immune response (Neal et al., 2013; Yazji et al., 2013; Lu et al., 2014; Nino et al., 2016), possesses an immature intestinal flora that has an increased composition of potentially pathogenic bacteria (Elgin et al., 2016), and contains an immature cohort of protective Paneth cells (Heida et al., 2016). In this mileau, based on the data we have presented, we propose that disruption of Paneth cell biology through inflammation (Brown et al., 2014) or other mechanisms can be the initiating factor that leads to a critical imbalance of the host-microbe axis that eventually ends in tissue damage and NEC. Further studies are needed to define this complex interaction.

## MATERIALS AND METHODS

### Mice

Mice were bred at The University of Iowa under standard conditions according to protocols approved by the Institutional Animal Care and Use Committee. All mice were dam-fed prior to experiments and, unless otherwise indicated, experiments were conducted with P14-P16 mice. All experiments had roughly equivalent numbers of male and female animals. On the day of experimentation, animals were separated from their mothers and maintained in a temperature- and humidity-controlled chamber. All mice were either wild-type C57BL/6J or on a C57BL/6J background, and founders were purchased from The Jackson Laboratory (Bar Harbor, ME). *PC-DTR* mice were generated by inserting a HA-tagged human diphtheria toxin receptor into the cryptdin-2 promoter on the surface of Paneth cells. The construct of this vector was a generous gift from Dr Jeff Gordon at Washington University (Garabedian et al., 1997). *PC-DTR* mice were generated in the University of Iowa Transgenic Mouse Core via pronuclear injection into FVB founders. PCR-positive mice were then backcrossed over 8 generations to a C57BL/6J background. Mice were screened for the presence of transgenes by extracting tail DNA and performing PCR using the following primers and conditions: Forward 5′-AACCCGGACCCTCCCACTGTAT-3′, Reverse 5′-ACCACGGCCAGGATGGTTGT-3′. The cycling conditions were denaturation (3 min at 95°C), annealing (1 min at 58°C) and extension (20 s at 72°C) for 34 cycles. *TLR4^−/−^* mice founders were a generous gift from Dr David Elliott, University of Iowa.

### NEC models

#### Dithizone-induced Paneth cell disruption

P14-P16 mice were given an intraperitoneal injection with either 50 mg/kg body weight dithizone (Sigma) dissolved in 20% NH_4_OH/EtOH solution, or an equivalent volume of NH_4_HO/EtOH buffer alone (pH 10.5, 100 µl concentrated NH_4_OH mixed in 500 µl 100% EtOH). Six hours after injection, mice were gastrically gavaged with 1×10^9^ CFU bacteria/kg body weight or an equivalent volume of sterile media (nutrient broth; ATCC) (Sherman et al., 2005; Zhang et al., 2012). Mice were monitored for 10 h after gavage and then euthanized for tissue harvesting.

#### Diphtheria-toxin-induced Paneth cell disruption

P14-P16 *PC-DTR* mice were given an intraperitoneal injection with either 40 ng/g body weight diphtheria toxin (2 µg/µl solution) in phosphate buffered saline (PBS), or an equivalent volume of PBS alone. Twenty-four hours after injection, mice were gavaged with 1×10^9^ CFU pathogen/kg body weight or an equivalent volume of sterile media (nutrient broth; ATCC) (Sherman et al., 2005; Zhang et al., 2012). Mice were monitored for 10 h after gavage and then euthanized for tissue harvesting.

#### Injury scoring

**Fig. 8.**
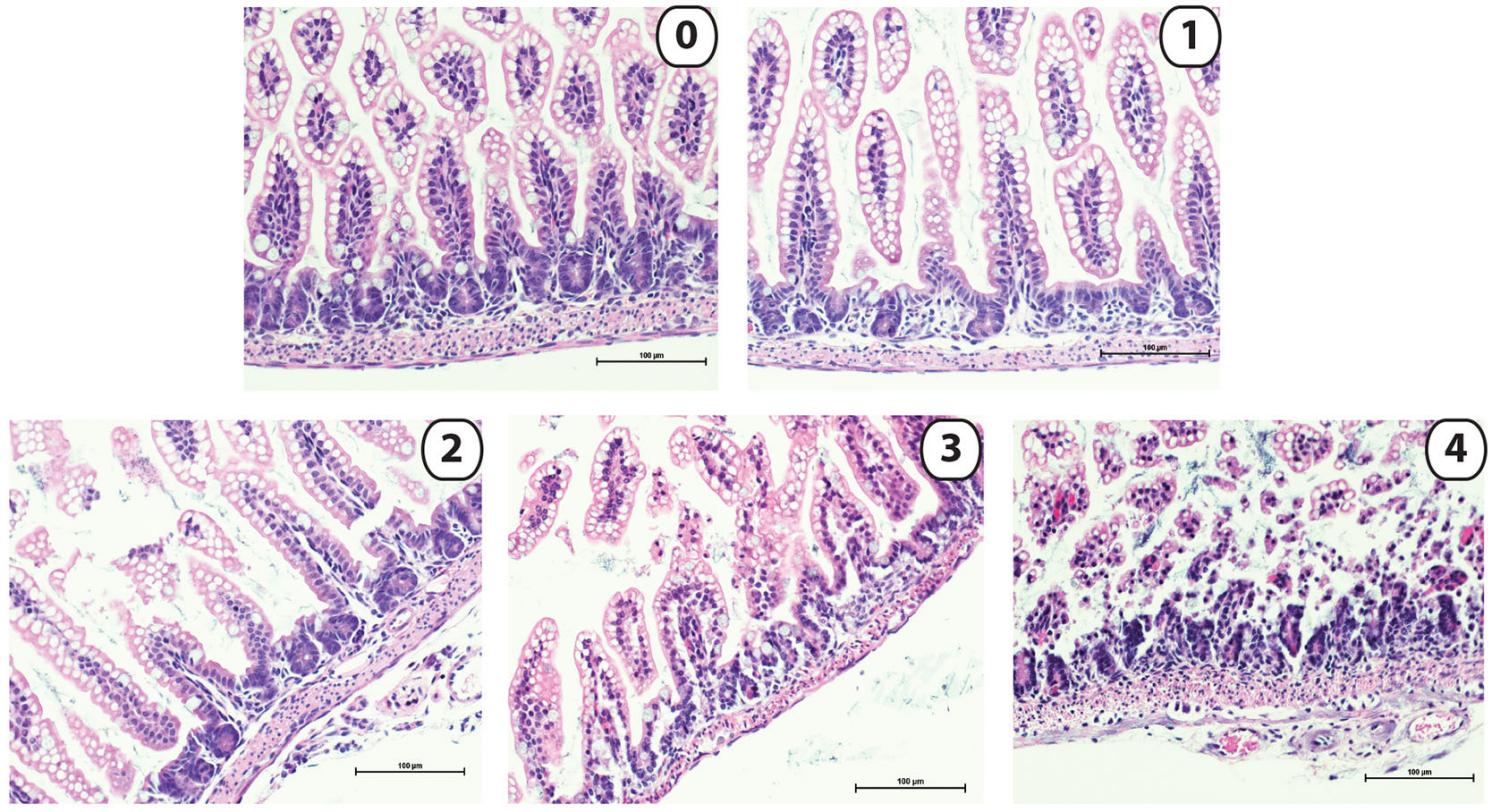
**Sample histology representing the NEC scoring scale.** Intestinal injury is scored on a standard Likert-like scale from 0-4, with a score of 2 or greater being significant for intestinal injury consistent with NEC. Histological changes in the mouse ileum in the continuum of NEC injury are illustrated by: grade 0, no injury; grade 1, mild separation of lamina propria; grade 2, moderate separation of sub-mucosa; grade 3, severe separation and/or edema in sub-mucosa; grade 4, transmural injury. Scale bars: 100 µm.

For all methods, mucosal injury was evaluated using a standard NEC scale by a single, blinded investigator (Musemeche et al., 1991; Dvorak et al., 2002; Maheshwari et al., 2011; Zhang et al., 2012) (Fig. 8). Injury was considered to be significant for scores greater than or equal to 2.

### Bacteria

Unless otherwise noted, all studies were performed using *K. pneumoniae* 10031 (ATCC, Manassas, VA). Prior to gavage, all bacteria were grown to log phase and optical density was performed to determine CFU quantity. All mice receiving bacteria were given 1×10^9^ CFUs pathogen/g body weight via a single gavage feed. *Klebsiella* *Zea mays* is a wild-type, GFP-labeled *Klebsiella* (Chelius and Triplett, 2000), and was a generous gift from Eric Triplett, University of Florida. *B.* *cereus* (George et al., 2003) was provided by P.S.’s lab. Heat-killed bacteria were grown to log phase and optical density obtained to verify equivalent CFUs as per our original protocol. Bacteria were subsequently exposed to a 67°C water bath for 20 min. Bacterial death was verified by plating samples of the resultant bacterial media on a sterile growth plate and placing the plate in a 37°C incubator for 24 h. Condensed conditioned media was generated from *Klebsiella* ATCC10031 by growing bacteria to log phase and determining CFU equivalence. Bacterial samples were then centrifuged at 5000 rpm (1677 ***g***) for 10 min and resultant supernatant was removed for experimental use. To guarantee that conditioned media was at least as potent as live bacteria, the supernatant was lyophilized to 10 times the original concentration, and then filtered through 45 μm filters to remove any residual bacteria. LPS concentrations were quantified using the e-toxate kit (Sigma-Aldrich). Cecal slurries were generated by dissecting ceca from control P14 mice using sterile instruments. Each cecum was placed in a sterile container and contents were collected using sterile forceps. Immediately following collection, cecal contents were pooled, suspended in 2 ml broth, and CFU was determined by optical density as above.

### Hematological variable quantification

Facial vein sampling without anesthesia was performed with a 3 mm Goldenrod animal lancet (Braintree Scientific, Braintree, MA) as previously described (White et al., 2016). In brief, blood was collected into 100 μl EDTA microvette tubes (Sarstedt, Numbrecht, Germany) and gently mixed to avoid platelet activation. Twelve microliters of whole blood were then transferred to a microcentrifuge tube containing 108 μl CellPak (Sysmex America, Lincolnshire, IL), as recommended by the manufacturer.

Samples were diluted 10-fold and placed on ice until analysis (<30 min from sampling). Analysis was performed using a Sysmex XT-200iV (Sysmex America, Lincolnshire, IL) analyzer in manual capillary mode.

### Cell lysates, PCR and western blotting

Ileal samples were homogenized using a TissueLyser LT (Qiagen), then cleared, and boiled as previously described (McElroy et al., 2008; Zhang et al., 2012; Brown et al., 2014). Proteins were separated by SDS-PAGE and transferred to nitrocellulose membranes. Membranes were incubated with anti-TLR4 and -pIKK primary antibody (Santa Cruz Biotechnology) overnight at 4°C, and incubated with secondary antibody (Cell Signaling) for 45 min. For mRNA quantification, ileal samples were homogenized as above and RNA was isolated using RNeasy Plus Mini Kit (Qiagen) according to the manufacturer's directions. RNA concentration and quality were determined using a NanoDrop 1000 Spectrophotometer (Thermo Fisher Scientific). qRT-PCR was performed using Taqman Fast Universal PCR Master Mix (2×) (Life Technologies) and Taqman Gene Expression Assays for cryptdin, lysozyme, TLR1-TLR7 and TLR9, ZO-1, occludin or E-cadherin primers (Life Technologies). qRT-PCR reactions were run in a C1000 Thermal Cycler (Eppendorf) using the CFX96 Real-Time PCR Detection System (Bio-Rad). Fold change in gene expression was determined by normalizing gene expression to β-actin in each sample. The 2^−ΔΔCT^ method was used to compare gene expression levels between samples.

### Paneth cell quantification

Ileal sections were stained with Alcian Blue/Periodic Acid Schiff stain (Sigma-Aldrich) as previously shown (McElroy et al., 2014). To minimize sectioning variability, all sections were obtained from the center of the intestinal sample and only areas with full villi were included. In each sample used for measurement, at least 3 distinct areas were counted to minimize sectioning variances. Cells were quantified with a 60× objective (600× total magnification) by a single, blinded investigator. Intestinal sections from at least 5 animals were analyzed for each experimental group and at least 100 crypts were counted per animal. All data were obtained using a Nikon NiU microscope using Nikon Elements software (Nikon).

### Cytokine analysis

Blood was obtained from the facial vein at the time of euthanasia (White et al., 2016). Whole-blood samples were placed on ice for 1 h then centrifuged at 7000 rpm (3287 ***g***) for 5 min to isolate serum. Cytokines were quantified using a Meso-Scale Discovery V-Plex assay (Meso-Scale, Gaithersburg, MD) according to the manufacturer's instructions. Plates were read on a Sector Imager 2400 at 620 nm.

### Serum zinc and glucose quantification

Blood glucose levels were monitored using a OneTouch Ultra glucose meter (Life Scan Inc., CA) at hourly intervals following dithizone injection. Zinc quantification was performed using a Quantichrom Zinc Assay Kit (BioAssay Systems DIZN-250). A 96-well plate was incubated for 30 min at room temperature and read at 425 nm (Molecular Devices, SpectraMax Plus). Zinc concentration was calculated from a standard curve.

### FITC-dextran analysis

Mice were gavaged with 0.6 mg/g body weight FITC-dextran (MW 40,000-70,000, Sigma-Aldrich) 9-10 h following dithizone injection or 3-4 h following bacterial gavage. Blood was obtained from sub-mandibular or facial vein puncture 4 h following gavage of FITC-dextran. Serum was isolated as described above. Samples were diluted to 1:16 and read using a fluorescence plate reader at 485-535 nm wavelength comparing serum samples to standard controls.

### Immunofluorescence

Samples were deparaffinized and rehydrated. To unmask antigens, citrate buffer (pH 6.0) was used in a Biocare Company Decloaking Unit at 110°C for 15 min followed by TBST washing (5 min×2) and blocking in 5% normal goat serum (Cell Signaling). Anti-ZO1 (Invitrogen, rabbit) 1:100 in Antibody Diluent (Cell Signaling, #8112) and anti-E-cadherin 1:100 (Dako, M3612, mouse) were then used. Sections were incubated with goat anti-rabbit Alexa Fluor 488 at 1:500 and goat anti-mouse Alexa Fluor 568 at 1:500 (Invitrogen) for 45 min at room temperature, washed 5 min×3 with PBS, and slides were mounted with hard-set fluorescence mounting medium (Vector Laboratories). Images were captured using confocal microscopy.

### Statistical analysis

All experiments were performed in at least triplicate and specific sample sizes are denoted in the Results. Non-parametric Kruskal–Wallis testing was performed to determine statistical significance using SAS v9.4, and GraphPad Prism v6. Significance was set as *P*<0.05 for all experiments. Experimental groups were compared to a control group that combined all sham animals from all trials to increase power and reduce animal numbers. Separate analysis of sham-treated animals was performed using non-parametric Kruskal–Wallis tests to determine that no statistical differences existed between these groups in any individual trials.
